# Leaf Orientation as Part of the Leaf Developmental Program in the Semi-Deciduous Shrub, *Cistus albidus* L.: Diurnal, Positional, and Photoprotective Effects During Winter

**DOI:** 10.3389/fpls.2019.00767

**Published:** 2019-06-19

**Authors:** Marina Pérez-Llorca, Andrea Casadesús, Maren Müller, Sergi Munné-Bosch

**Affiliations:** ^1^ Department of Evolutionary Biology, Ecology and Environmental Sciences, Faculty of Biology, University of Barcelona, Barcelona, Spain; ^2^ Department of Evolutionary Biology, Ecology and Environmental Sciences, Faculty of Biology, Institut de Recerca de la Biodiversitat, University of Barcelona, Barcelona, Spain

**Keywords:** abiotic stress, *Cistus albidus* L., leaf orientation, leaf positional effects, low temperature stress, melatonin, photoinhibition, photoprotection

## Abstract

Mediterranean ecosystems harbor a very important part of Earth’s biodiversity, and they are a conservation priority due to the effects of global change. Here, we examined the performance of the semi-deciduous shrub *Cistus albidus* under Mediterranean conditions during winter, including changes in leaf angle governed by diurnal, seasonal, and positional effects and their relationship with winter photoinhibition and photoprotection. We found marked diurnal variations in leaf angle during the day in autumn, which disappeared as the photoperiod shortened during winter due to a progressive decrease in the predawn leaf angle from November to January. During this period, auxin contents decreased, while those of melatonin increased, and the *F*_v_/*F*_m_ ratio, chlorophyll, and tocopherol contents kept unaltered, thus indicating the absence of photoinhibitory damage. A marked decrease in the leaf angle toward the shoot apex occurred during winter, which was associated with slightly higher *F*_v_/*F*_m_ ratios. An analysis of the inter-individual variability and sun orientation effects on leaf movements in a population growing in the Montserrat mountains revealed a very marked variability of 46.8% in the leaf angle, while *F*_v_/*F*_m_ ratio showed a variation of 7.5% only. West orientation, which was associated with reduced leaf temperatures, but with no differences in the photosynthetic photon flux density, led to enhanced tocopherol contents, while leaf angle, *F*_v_/*F*_m_ ratio, chlorophyll, auxin, and melatonin contents kept unaltered. It is concluded that (1) *C. albidus* has very effective and fine-regulated photoprotection mechanisms, including an adequate orientation of decussate leaves as part of the developmental program, (2) leaf angle is modulated on a diurnal and seasonal basis, thus contributing to prevent photoinhibition as a first line of defense, and (3) enhanced tocopherol contents help withstand combined high light with low temperature stress in *C. albidus* growing at high elevation.

## Introduction

The Mediterranean basin is one of the most unique biomes that harbors an important portion of Earth’s plant biodiversity. Mediterranean ecosystems have been described as especially delicate upon the predicted extreme climatic events due to climate change (Peñuelas et al., 2017), and given that there is already a constant loss of biodiversity (Butchart et al., 2010), Mediterranean plant species are a conservation priority (Bellard et al., 2014). Light has been classically considered as one of the main stressors in Mediterranean-type ecosystems for plants (Joffre et al., 1999; Karavatas and Manetas, 1999), particularly excess of light, which together with drought (typically occurring during summer) or low temperatures (during winter) can cause considerable damage to the photosynthetic apparatus (Martínez-Ferri et al., 2004; Fernández-Marín et al., 2017).

Mediterranean plants have evolved different photoprotection mechanisms to avoid this excess of light, building a first line of defense. There are structural adaptations to avoid excess of light absorption such as tomentosus leaves that reflect light (Ehleringer and Björkman, 1978), chloroplast movements during the day (Haupt and Scheuerlein, 1990), changes in leaf orientation (Ehleringer and Comstock, 1987), paraheliotropism (Lambers et al., 2008), and a phyllotaxis with an opposite disposition of leaves (Brites and Valladares, 2005). In the case of leaf paraheliotropism, for instance, not all plants species exhibit it but it has developed in multiple lineages, including the well-known common bean (*Phaseolus vulgaris* L.), where this movement is triggered by an endogenous circadian oscillator and light-induced signals that lead to turgor-dependent changes in the pulvinus, which is located at the juncture of the leaf base and the petiole (Mayer et al., 1985). In several Mediterranean plants, such as the white-leaved rockrose (*Cistus albidus* L.), reduced leaf angles occur as part of the developmental program with opposite and decussate leaves (without petioles) increasing their leaf angle as they develop at the shoot apex and progressively occupy more distal positions (De Bolòs, 1993).

Once light has gone through the structural photoprotection mechanisms, it is absorbed by leaves and penetrates the chloroplasts, plants activate a second line of defense to counteract excess solar radiation. The first and one of the most flexible mechanisms to counteract the excess excitation energy and, consequently, photo-oxidative stress in chloroplasts is the xantophyll cycle (Demmig-Adams and Adams, 2006). However, if the excited triple chlorophyll (^3^Chl*) derived from excited chlorophyll a (^1^Chl*) is not quenched by the non-photochemical quenching (NPQ) in the photosystem II, this can lead to the production of singlet oxygen (^1^O_2_) and ultimately to the oxidation of lipids and other molecules in the chloroplast. At the same time, O_2_ can also be over-reduced by the electrons originated in the photosystem I to superoxide radicals (O_2_^−^) and then form hydrogen peroxide (H_2_O_2_) and finally hydroxyl radical (OH^−^), which is highly reactive (Asada, 2006). The joint effects of these ROS can cause photoinhibition, and if this is sustained, plants will experience photo-oxidative damage (Takahashi and Badger, 2011). This can be prevented by the concerted action of a myriad of antioxidant compounds. Among them, lipophilic compounds, such as tocopherols (vitamin E), have been shown to play a major role in photoprotection (Havaux et al., 2005) acting as a sentinel for stress sensing and signaling (Munné-Bosch, 2019). On the other hand, melatonin, a tryptophan-derived compound (sharing part of the biosynthesis pathway with the phytohormone indole-3-acetic acid, Pérez-Llorca et al., 2019) has been postulated as a putative antioxidant and photoprotective compound in plants (Ding et al., 2017). However, it is still not fully elucidated whether or not melatonin always acts directly as an antioxidant or indirectly through its modulatory effects on gene expression (Arnao and Hernández-Ruiz, 2019).

The aim of this study was to get new insights into the performance of the semi-deciduous shrub, *C. albidus* during high light stress and suboptimal (low) temperatures during the Mediterranean winter, with a particular emphasis on the causes and consequences of changes in leaf orientation as part of the developmental program. We hypothesized that leaf movements may be governed on a diurnal and seasonal basis and that they may significantly influence the extent of photoinhibition and photoprotective demand of leaves during winter. To test this hypothesis, we measured (1) leaf angles during predawn and midday from November to January, (2) leaf positional effects on leaf angles during winter (January), and (3) the inter-individual variability and sun orientation influence on leaf angles and the *F*_v_/*F*_m_ ratio in a natural population growing at 1,100 m.a.s.l., which was exposed to a combination of high light and low temperatures. Furthermore, we studied the endogenous variations in melatonin in relation to a biosynthesis-related phytohormone (auxin) and a well-known chloroplastic antioxidant (α-tocopherol) to get new insights into the possible protective effects of this compound against environmental stress in plants.

## Materials and Methods

### Experimental Design and Sampling

Three independent, complementary experiments were performed using white-leaved rockrose (*Cistus albidus* L.). The first experiment was focused on the study of diurnal variations in leaf angle from autumn to winter, including measurements at predawn (1 h before sunrise) and midday (at maximum diurnal photosynthetic photon flux density; PPFD). The samplings were performed on November 6 and December 4, 2017 (autumn), and January 16, 2018 (winter) in a population growing in the experimental garden of the Faculty of Biology at the University of Barcelona (41.384 N, 2.119E, 59 m.a.s.l.). All measurements from this study were made using leaves situated on the second whorl of the plant (second leaf position) from the top of shoots. The second experiment was focused on the study of leaf positional effects on leaf angle during winter. Leaves situated on the first, second, third, fourth, and fifth whorl (designated here, arbitrarily, as positions 1–5 and corresponding to two different leaf dispositions on the stem) from the top of shoots of the same plants as those used for the first experiment were selected for measurements during January 16, 2018 at midday (at maximum diurnal PPFD). The third experiment focused on sun orientation and inter-individual driven variability in the leaf angle of a natural population in the Natural Park of the Montserrat mountains, Spain (41.586 N–1.830 E, 1100 m.a.s.l.) during March 22, 2018. A stressful cold and sunny day was selected for measurements, with maximum and minimum air temperatures during the day of 14.7°C and −0.2°C, respectively (mean relative humidity was 30% and maximum diurnal PPFD 1570 μmol m^−2^ s^−1^, while no precipitation during the day was recorded). Sixty individuals of the same population, but with different sun orientations, were sampled; 30 were East oriented and 30 were West oriented (with an average leaf temperature of 11°C and 19°C, respectively, with no difference in the average relative humidity and maximum diurnal PPFD). All measurements for this study were made using leaves situated on the second whorl (position 2) from the top of shoots in all individuals. Details of soil characteristics of both sites (experimental garden from the University of Barcelona and the Montserrat mountains) can be found in Müller et al. (2013).

For the three experiments, all samplings at each time point were performed using two leaves of the same whorl per shrub from same-aged individuals that were randomly selected at the beginning of the study. Aside from leaf angle, which was described with the zenith and the azimuth angles of the surface normal to a small flat plate (Itakura and Hosoi, 2018), leaf biomass, leaf mass per area ratio, leaf hydration, chlorophyll contents, *F*_v_/*F*_m_ ratio, indole-3-acetic acid (IAA), melatonin, and α-tocopherol contents were measured as described below. For all biochemical analyses, samples were immediately snap frozen in liquid nitrogen and stored at −80°C until subsequent processing in the laboratory.

### Environmental Conditions, Photoinhibition, and Other Physiological Markers

PPFD, leaf temperature, and the *F*_v_/*F*_m_ ratio (after 1 h of darkness), the latter used as an indicator of photoinhibition (Bolhar-Nordenkampf et al., 1991), were measured with a Mini-PAM II (Photosynthesis Yield Analyzer, Walz, Germany) *in situ*. Leaves were immediately weighed to estimate leaf biomass and leaf area was measured using a flatbed scanner (model Officejet Pro 8610, HP, California, USA). Then, dry mass was estimated by weighing the sample after oven drying it at 65°C to constant weight. Leaf hydration (H) was calculated as (fresh mass-dry mass)/dry mass, and leaf mass per area (LMA) was calculated as dry mass/area.

### Biochemical Analyses

The chlorophyll a + b content of leaves was determined spectrophotometrically in methanol extracts using the equations described by Lichtenthaler (1983). Melatonin and auxin contents were determined by ultrahigh-performance liquid chromatography coupled to tandem mass spectrometry (UHPLC-MS/MS), and α-tocopherol was determined by high-performance liquid chromatography (HPLC). In short, 50 mg per sample was extracted with 250 μl of cold methanol using ultrasonication and vortexing (Branson 2510 ultrasonic cleaner, Bransonic, Danbury, CT, USA) for 30 min. Deuterium-labeled indole-3-acetic and melatonin were then added, and after centrifugation at 12,000 rpm for 10 min at 4°C, the pellet was re-extracted using the same procedure. Supernatants were pooled and filtered through a 0.22-μm PTFE filter (Waters, Milford, MA, USA) before UHPLC-MS/MS and HPLC analyses. Indole-3-acetic and melatonin contents were analyzed by using UHPLC-ESI-MS/MS as described in Müller and Munné-Bosch (2011). Quantification was made considering recovery rates for each sample by using deuterium-labeled internal standards. α-Tocopherol contents were determined by using a normal-phase HPLC system coupled to a fluorescent detector as described in Cela et al. (2011). α-Tocopherol was identified by co-elution with an authentic standard (Sigma-Aldrich, Steinheim, Germany) and quantified using a calibration curve.

### Statistical Analysis

To assess the combined effects of month (“November,” “December,” “January”) and daytime (“Predawn,” “Midday”) on leaf angle, leaf biomass, leaf mass per area, hydration, chlorophyll a + b, *F*_v_/*F*_m_ ratio, IAA, melatonin, and α-tocopherol, we used a linear mixed model (LMM). Different combinations of “Month,” “Daytime,” and their interaction were tested as fixed terms using Akaike information criterion (AIC), i.e., the model that best fitted the data should have the lowest AIC value. “Plant” was included in all models as a random term to account for repeated measures (Zuur et al., 2009). Models were fitted with maximum likelihood (ML) for model comparison. Final models were fitted using restricted maximum likelihood (REML). Additionally, when “Daytime” had a significant effect, an LMM with “Daytime” as an explanatory variable was used to test for significant differences within each month. To assess significant differences within leaf position on the response variables above mentioned, we used a LMM where “Leaf position” was fitted as the fixed term and “Plant” as a random term. To assess the effect of sun orientation in the Montserrat natural-growing population, a linear regression was used with “Orientation” as explanatory variable. In all models, the *p* of the main fixed effects were estimated using conditional *F*-tests (generally, ANOVA type II or ANOVA type III when an interaction term was included), and when main effects were significant, multiple comparisons between “Month” and “Daytime,” or “Leaf position” levels, were tested with the Tukey HSD post hoc test. All models were validated by visually checking the distribution of residuals for normality and homoscedasticity (Zuur et al., 2009). Before analyses, leaf angle, chlorophyll a + b, IAA, and melatonin were log_10_-transformed; leaf mass per area and α-tocopherol were square root-transformed; and *F*_v_/*F*_m_ ratio was logit-transformed. Finally, to explore possible relationships between all response variables, we used the Spearman’s rank correlation test. All analyses were performed using the computing environment R (R Core Team 2019).

## Results

### Leaf Angle is Modulated on a Diurnal and Seasonal Basis in *C. albidus*

**Figure 1:**
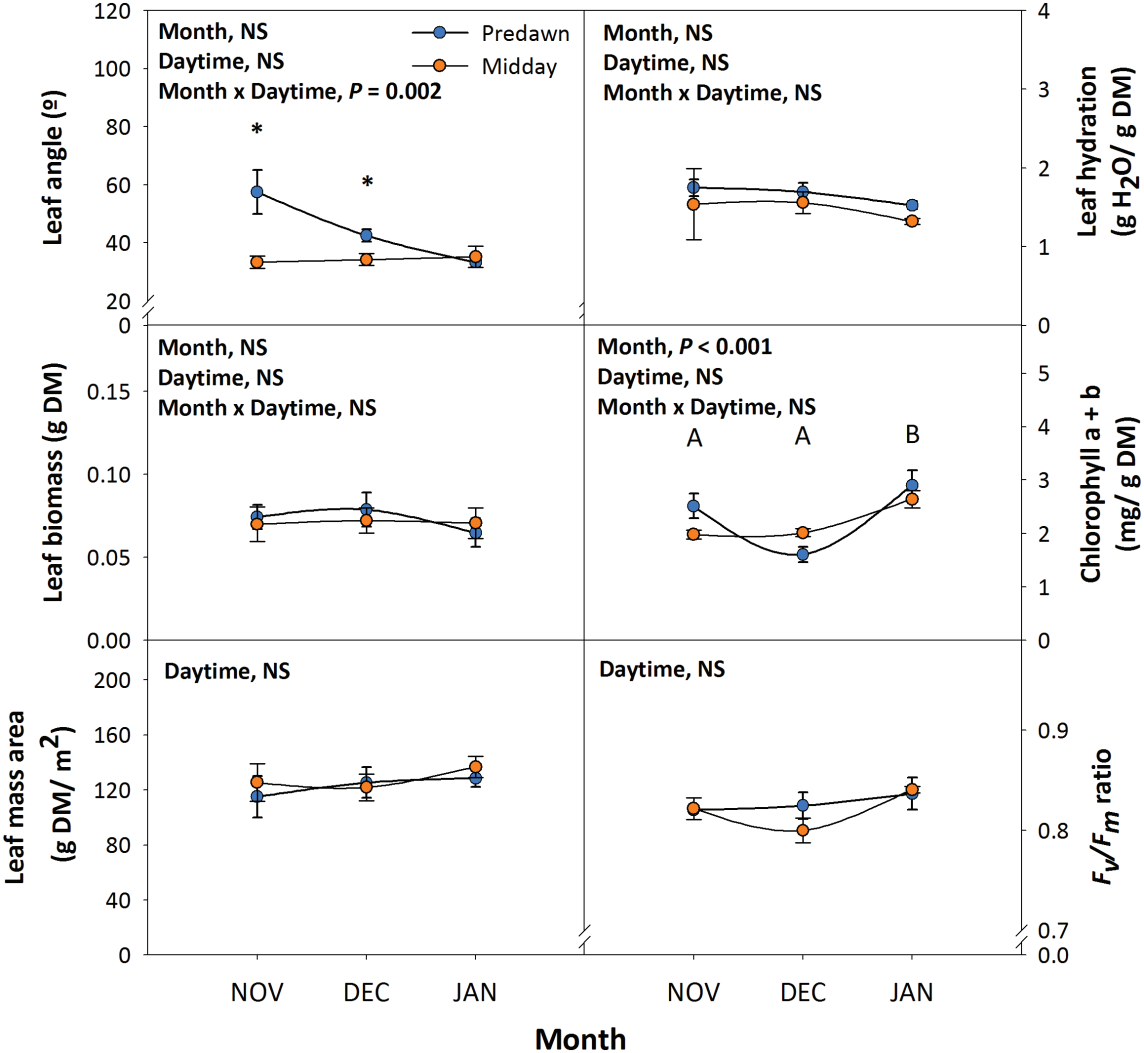
Diurnal and seasonal changes in leaf orientation in the Mediterranean shrub, *C. albidus*. A comparison of leaf angle, biomass, mass per area ratio, hydration, chlorophyll contents, and the *F*_v_/*F*_m_ ratio between predawn and midday from autumn (November) to winter (January) in plants growing at the experimental garden of the University of Barcelona is shown. Data correspond to leaf position 2 from the apex (see photograph in Figure 3 for details) and represent the mean ± SE of *n* = 6 individuals. After model selection, significant differences in “Month” and “Daytime” were tested by conditional Fisher tests (*p* ≤ 0.05). Different capital letters indicate significant differences in Tukey’s HSD multiple comparison test (*p* ≤ 0.05) with all data. Asterisks indicate significant differences in Tukey’s HSD multiple comparison test in “Daytime” within “Month”. NS, not significant; DM, dry matter.

Leaf angle measurements at predawn and midday during 6th November, 4th December, and 16th January revealed significant diurnal variations in leaf orientation. Leaves had a reduced angle during the day in autumn (November and December) but not in winter (January), when maximum orientation parallel to sun’s rays was attained already at predawn (Figure 1). It is interesting to note that despite diurnal variations in leaf orientation in autumn, maximum orientation parallel to the sunbeam was always attained at midday, with leaf angles around 35°, irrespective of the day of measurements (Figure 1). Diurnal PPFD during this period changed from non-detectable values at predawn to values ranging between 892 and 989 μmol m^−2^ s^−1^ at midday, while the photoperiod was shortened from 10.3 h on 6th November to 9.5 h on 16th January. Leaf biomass, leaf mass per area ratio, and leaf hydration kept unaltered throughout the study (Figure 1). Despite chlorophyll contents decreased slightly on 4th December at predawn, which might be associated with temperatures reaching minimum values of 3.9°C (Table 1), the *F*_v_/*F*_m_ ratio, an indicator of photoinhibition, kept unaltered throughout the study (Figure 1).

**Table 1:** Environmental conditions during the sampling days both in the Experimental garden at the Faculty of Biology of the University of Barcelona and in the Natural Park of the Montserrat mountains.

	Min *T*^e^ (°C)	Max *T*^e^ (°C)	Mean *T*^e^ (°C)	Mean RH (%)	Mean PPFD (μmol/m^2^ s^−1^)	Max PPFD (μmol/m^2^ s^−1^)
Experimental garden						
6th November	10.0	17.8	13.2 ± 0.34	33.56 ± 2.05	671 ± 79.45	989
4th December	3.9	15.2	9.2 ± 0.45	41 ± 1.37	459 ± 84.80	892
16th January	7.9	18.0	12.8 ± 0.46	58.2 ± 0.67	330 ± 85.25	936
Montserrat mountains						
22nd March	−0.2	14.7	4.18 ± 0.93	51.09 ± 3.53	510 ± 68.35	2531

*Min *T*^e^, minimum temperature; Max *T*^e^, maximum temperature; Mean *T*^e^, mean temperature; Mean RH, mean relative humidity, Mean PPFD, mean daily photosynthetic photon flux density; Max PPFD, maximum photosynthetic photon flux density. Climate data were obtained from a public database (*Servei meteorològic de Catalunya*)*.

**Figure 2:**
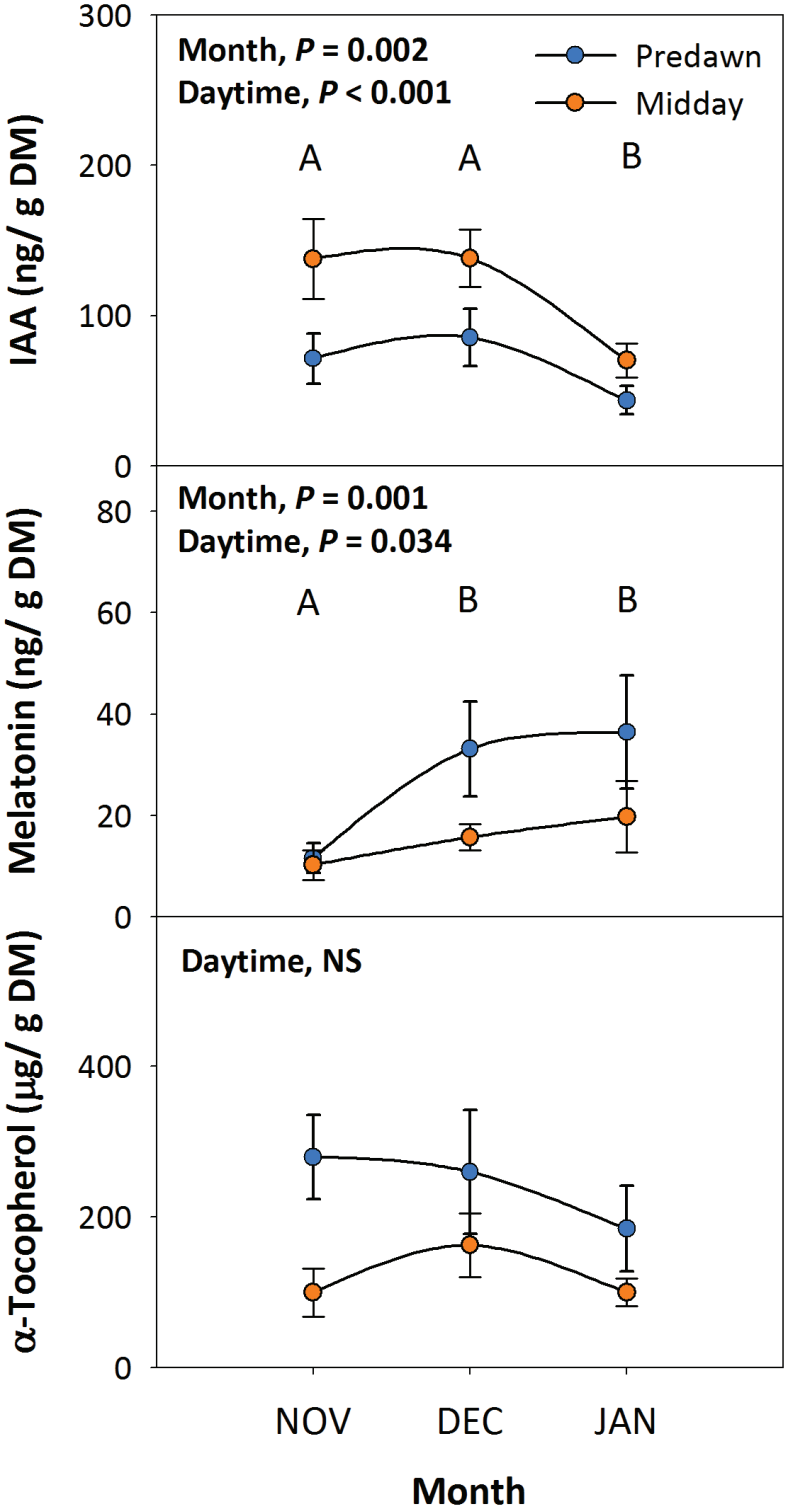
Diurnal and seasonal changes in endogenous melatonin contents in the Mediterranean shrub, *C. albidus*. A comparison between melatonin contents relative to those of the biosynthesis-related phytohormone auxin and the well-known chloroplastic antioxidant α-tocopherol between predawn and midday from autumn (November) to winter (January) in plants growing at the experimental garden of the University of Barcelona is shown. Data correspond to leaf position 2 from the apex (see photograph in Figure 3 for details) and represent the mean ± SE of *n* = 6 individuals. After model selection, significant differences in “Month” and “Daytime” were tested by conditional Fisher tests (*p* ≤ 0.05). Different capital letters indicate significant differences in Tukey’s HSD multiple comparison test (*p* ≤ 0.05) with all data. Asterisks indicate significant differences in Tukey’s HSD multiple comparison test in “Daytime” within “Month”. NS, not significant; DM, dry matter.

To investigate the possible protective effects of melatonin against environmental stress in plants, we studied the endogenous variations in melatonin in relation to its biosynthesis-related phytohormone auxin (indole-3-acetic acid, IAA) and a well-known chloroplastic antioxidant (α-tocopherol). Endogenous auxin contents decreased as the season progressed from autumn to winter, and values at midday were higher than those at predawn, whereas melatonin contents increased slightly during the same period with higher values at predawn than at midday (Figure 2). While melatonin contents were on the same order of magnitude than those of auxin, they were, however, four orders of magnitude smaller than those of α-tocopherol, the levels of which kept unaltered throughout the study (Figure 2).

### Positional Effects on Leaf Orientation in *C. albidus*

**Figure 3:**
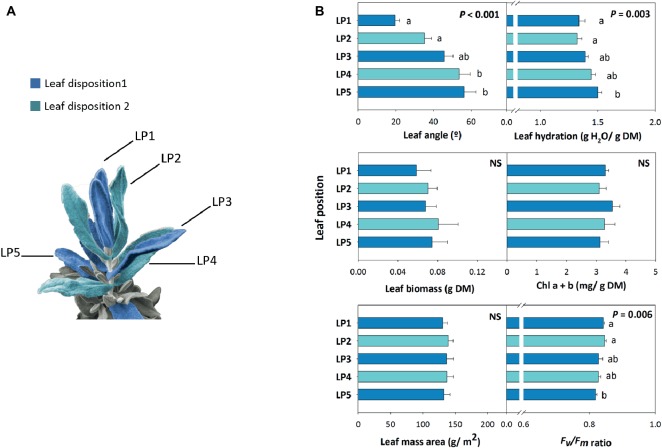
Positional effects in leaf orientation in the Mediterranean shrub, *C. albidus*. **(A)** Schematic representation of the disposition on the shoot (Leaf disposition 1 and 2 shown in dark blue and light blue, respectively) of the five leaf positions (LP1…LP5). **(B)** A comparison between leaf positions on leaf angle, biomass, mass per area ratio, hydration, chlorophyll contents, and the *F*_v_/*F*_m_ ratio in plants growing at the experimental garden of the University of Barcelona in January at midday is shown. Data represent the mean ± SE of *n* = 6 individuals. Significant differences in “Leaf position” were tested by conditional Fisher tests (*p* ≤ 0.05), and different letters indicate significant differences in Tukey’s HSD multiple comparison test (*p* ≤ 0.05). NS, not significant; DM, dry matter.

Leaf angle measurements at midday during 16th January revealed a marked positional effect on leaf orientation, with opposite, decussate leaves progressively showing an increased leaf angle from top to bottom of the shoot (Figure 3). Leaf biomass and leaf mass per area ratio kept unaltered with leaf position, while leaf hydration slightly increased by a 20% from leaf position 1 to 5 (Figure 3). Despite chlorophyll contents kept unaltered with leaf position, the *F*_v_/*F*_m_ ratio decreased slightly with leaf opening. It is noteworthy, however, that this ratio kept always above 0.80 in all studied leaf positions (Figure 3).

**Figure 4:**
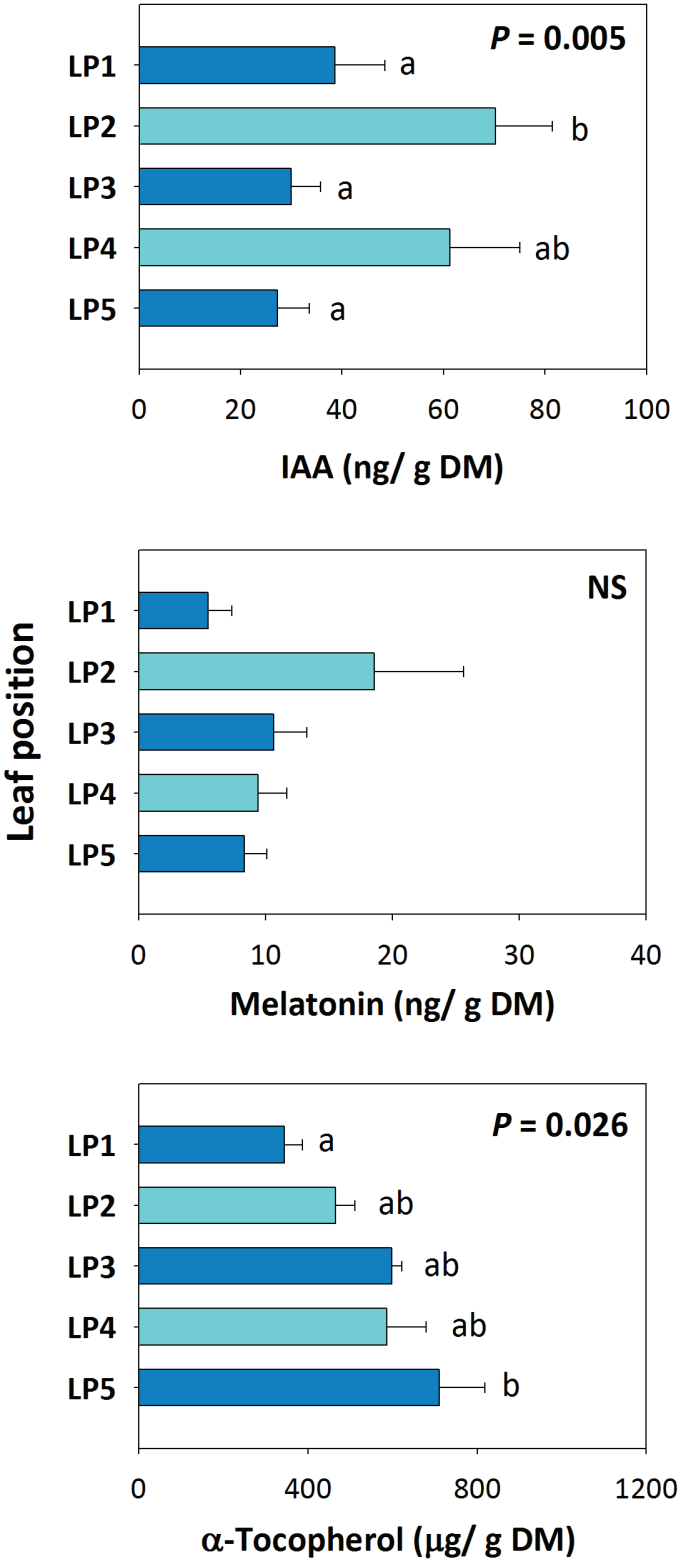
Positional effects in endogenous melatonin contents in the Mediterranean shrub, *C. albidus*. A comparison between melatonin contents relative to those of the biosynthesis-related phytohormone auxin and the well-known chloroplastic antioxidant α-tocopherol in plants growing at the experimental garden of the University of Barcelona during January at midday is shown. Data represent the mean ± SE of *n* = 6 individuals. Significant differences in “Leaf position” were tested by conditional Fisher tests (*p* ≤ 0.05), and different letters indicate significant differences in Tukey’s HSD multiple comparison test (*p* ≤ 0.05). NS, not significant; DM, dry matter.

Positional effects on the endogenous variations in melatonin in relation to auxin and α-tocopherol revealed that, although endogenous auxin contents were different between even and odd whorls, neither auxin nor melatonin contents were altered by increased leaf angle from top to bottom (Figure 4). In contrast, the contents of α-tocopherol increased concomitantly with less steep leaf angles from top to bottom (Figure 4).

### Influence of Sun Orientation and Inter-Individual Variability on Leaf Angle in *C. albidus*

**Figure 5:**
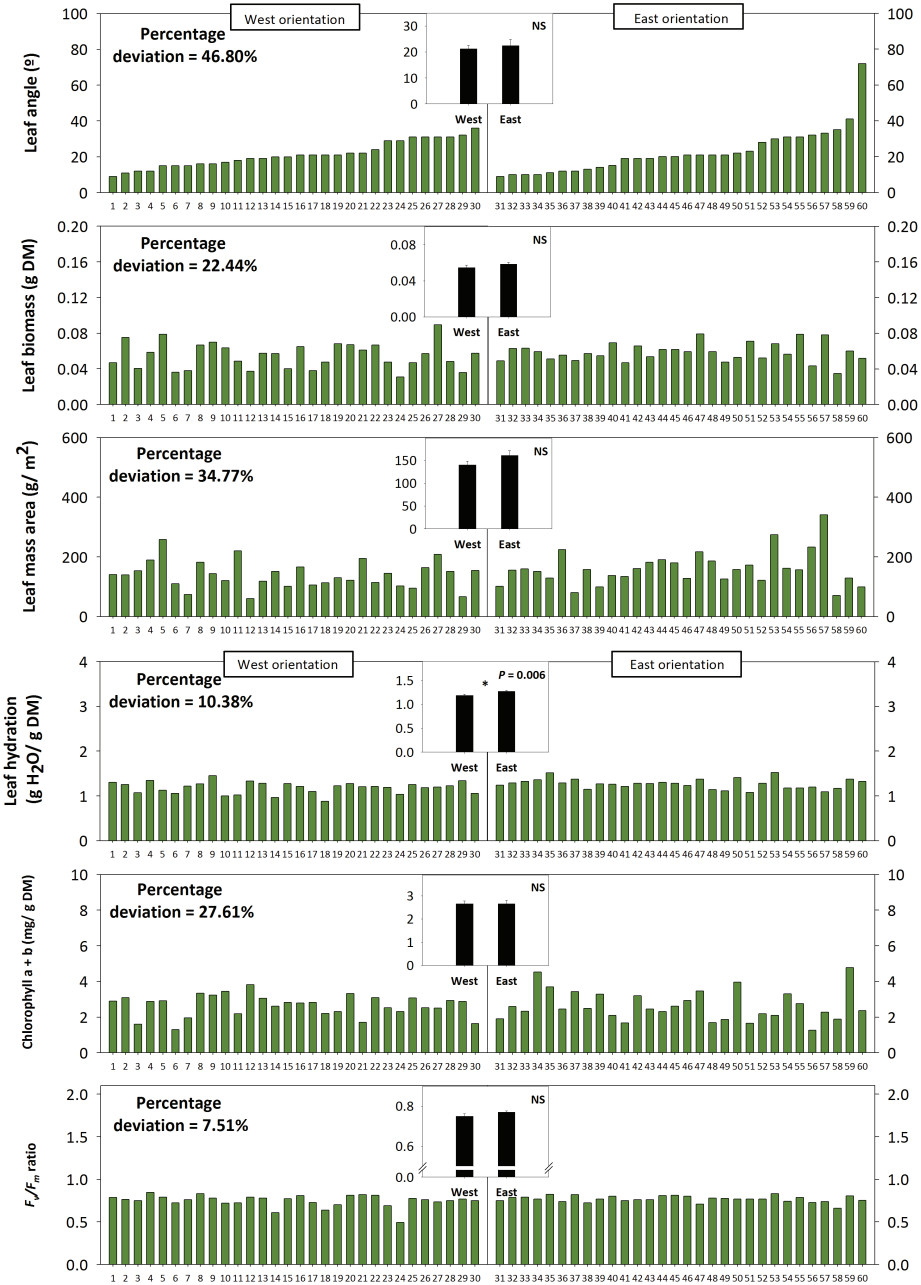
Effects of sun orientation and inter-individual variability on leaf orientation in the Mediterranean shrub, *C. albidus*. A comparison between leaf angle, biomass, mass per area ratio, hydration, chlorophyll contents, and the *F*_v_/*F*_m_ ratio in a *C. albidus* population growing at 1,100 m.a.s.l. in the Natural Park of the Montserrat Mountains with different sun orientation (East orientation, *n* = 30 individuals; West orientation, *n* = 30 individuals) during winter (22nd March 2018) is shown. Data correspond to leaf position 2 from the apex (see photograph in Figure 3 for details) for all studied individuals. The number of the individual (ordered from lower to higher leaf angle) is indicated in the x-axis. Percent deviation for every variable is given as (SD/Population mean) × 100, where SD corresponds to standard deviation. The mean ± SE for each sun orientation is shown in the graphs located in the inlets. Significant differences between groups were tested by a one-way analysis of variance (ANOVA, *p* ≤ 0.05) and indicated with an asterisk. NS, not significant.

**Figure 6:**
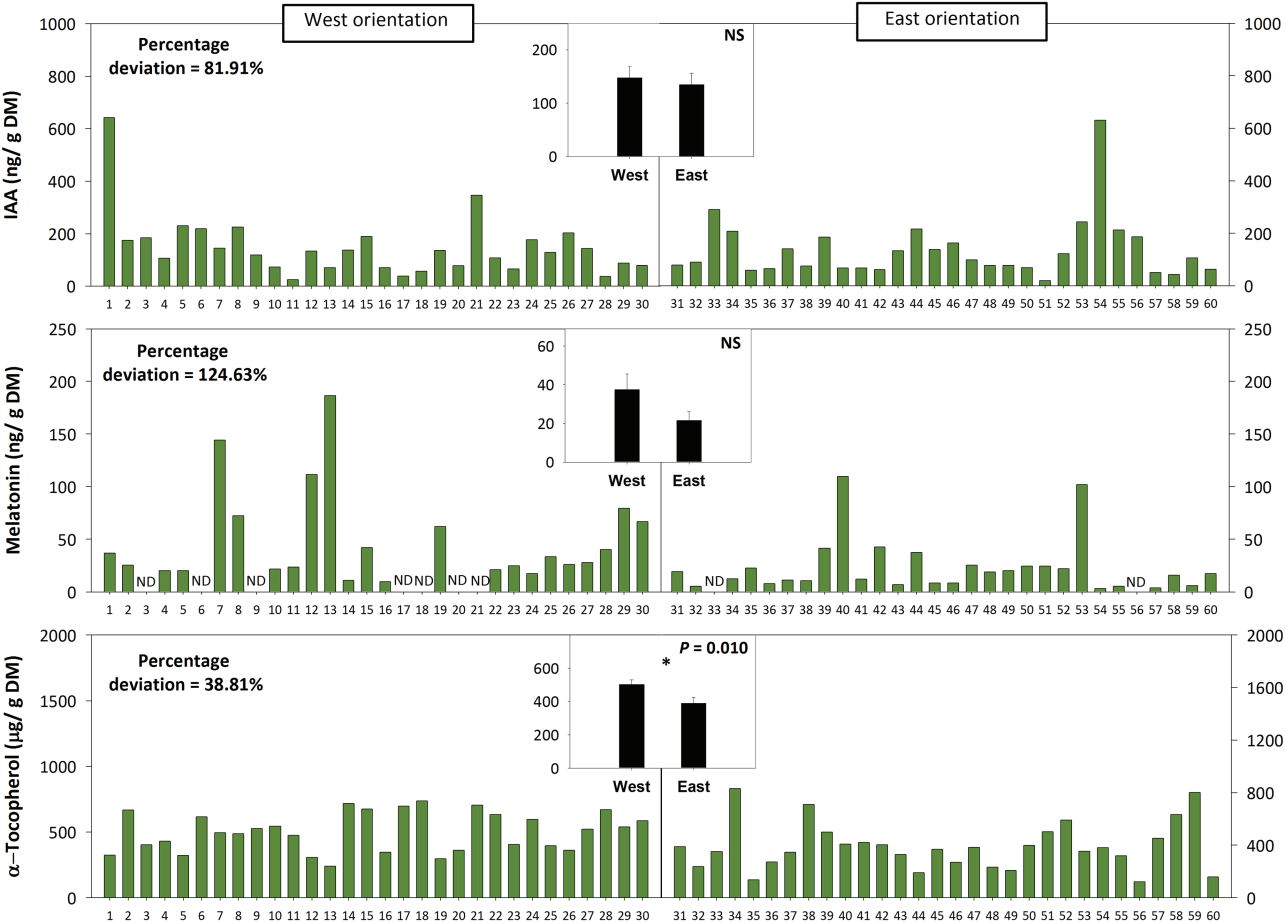
Effects of sun orientation and inter-individual variability on endogenous melatonin contents in the Mediterranean shrub, *C. albidus*. A comparison of melatonin contents relative to those of the biosynthesis-related phytohormone auxin and the well-known chloroplastic antioxidant α-tocopherol in a *C. albidus* population growing at 1,100 m.a.s.l. in the Natural Park of the Montserrat Mountains with different sun orientation (East orientation, *n* = 30 individuals, West orientation, *n* = 30 individuals) during winter (22nd March 2018) is shown. Data correspond to leaf position 2 from the apex (see photograph in Figure 3 for details) for all studied individuals. The number of the individual (ordered from lower to higher leaf angle) is indicated in the x-axis. Percent deviation for every variable is given as (SD/population mean) × 100, where SD corresponds to standard deviation. The mean ± SE for each sun orientation is shown in the graphs located in the inlets. Significant differences between groups were tested by a one-way analysis of variance (ANOVA, *p* ≤ 0.05) and indicated with an asterisk. NS, not significant; DM, dry matter.

Leaf angle measurements at midday during 22nd March in a natural population of *C. albidus* growing in the Montserrat mountains at 1,100 m.a.s.l. revealed a strong variability – quantified as percentage deviation (standard deviation/population mean) × 100 – on leaf orientation. Leaf at position 2 from top of the shoot from 60 individuals showed angles ranging from 10 to 80° (Figure 5). The variation in the leaf angle of both West-oriented and East-oriented individuals was much higher than that of leaf biomass, leaf mass per area ratio, leaf hydration, chlorophyll contents, and the *F*_v_/*F*_m_ ratio (Figure 5). Indeed, variability in leaf angle was 46.8%, while that of the *F*_v_/*F*_m_ ratio was 7.5% only. West orientation, which was associated with markedly reduced leaf temperatures and slightly lower leaf water contents compared to the East – but with no differences in the PPFD (Figure 5, see also materials and methods for details) –, led to enhanced tocopherol contents, while leaf angle, *F*_v_/*F*_m_ ratios, chlorophyll, IAA, and melatonin contents kept unaltered (Figures 5, 6).

## Discussion

Several semi-deciduous shrubs that represent an important hotspot of the Mediterranean flora, such as the white-leaved rockrose (*Cistus albidus* L.), show changes in leaf angle as part of their developmental program with opposite and decussate leaves without petioles increasing their leaf angle as they develop from top to bottom of the shoot apex. An enhanced leaf angle in more distal positions from the uppermost side of the shoot progressively leads to a leaf orientation more perpendicular to the sun’s rays, which results in an increased exposure to high light. Therefore, the youngest leaves, which develop later in time, are the ones with smaller leaf angles and, therefore, enhanced photoprotection due to reduced light incidence. There are several studies in *Cistus* spp. that report that higher leaf inclinations (i.e., steeper leaf angles) prevent photoinhibition and confer a higher stress tolerance during summer drought (*C. incanus*, Grattani and Bombelli, 2000; *C. monspeliensis*, Werner et al., 1999, 2001; *C. ladanifer*, Brites and Valladares, 2005; *C. salviifolius* and *C. monspeliensis*, Puglielli et al., 2017). However, the implications of these leaf movements to prevent winter photoinhibition have not been considered in detail thus far, with the exception of the study performed by Oliveira and Peñuelas (2002), which showed reduced cold-induced photoinhibition in leaves with smaller leaf angles and therefore less sun-exposed leaves in *C. albidus*. Here, we show that changes in leaf angle are governed by leaf position, as part of the leaf developmental program of shoots, and by environmental conditions both diurnally and seasonally, all providing a first line of defense to prevent photoinhibition in this plant species. At the same time, we also show that, in nature, the variability in leaf angles is so high that other mechanisms operating at the chloroplast level, such as an enhanced vitamin E biosynthesis, are required for counteracting the potential harmful effects of combined high light with low temperatures in populations growing at high elevation.

Leaf orientation was not only influenced by the intrinsic shoot developmental program in *C. albidus,* but it also changed on a diurnal and seasonal basis, both factors interacting between them. As it occurs with paraheliotropism in some legumes (Mayer et al., 1985; Raeini-Sarjaz, 2011), the leaf angle seemed to be governed to some extent, at least, by circadian rhythms in *C. albidus* since leaves had a steeper angle at midday compared to predawn, particularly in autumn. However, the absence of a petiole and most probably of a pulvinus in *C. albidus* leaves suggests that this movement is not as “active” as in some legumes such as common bean (Lambers et al., 2008), but the result of a more “passive” process associated with the plant developmental program. However, the fact that leaves move on a diurnal basis indicates that this leaf movement responds to light and can, therefore, be considered a type of paraheliotropism, as recently suggested by Puglielli et al. (2017) and *sensu* Häder and Lebert (2001).

Enhanced tocopherol contents were observed in the West-oriented individuals compared to the East-oriented ones, which might help withstand combined high light with low temperature stress in *C. albidus* growing at high elevation. Mean leaf temperature during samplings in the West orientation was 11°C, in contrast to the 19°C in East orientation, while the PPFD did not differ between both orientations. Reduced leaf temperature in the West led to a slightly reduced leaf hydration, which may be associated with dehydration caused by chilling (Hussain et al., 2018). In turn, low temperatures have been shown to increase α-tocopherol contents in other plant species as a mean to counteract ROS production and allow stabilization of membranes (Munné-Bosch, 2005; El Kayal et al., 2006). Indeed, tocopherols may not only protect the photosynthetic apparatus during plant exposure to extreme temperatures but also influence retrograde signaling, thus acting in stress sensing and signaling (Fang et al., 2019; Munné-Bosch, 2019). Interestingly, the *F*_v_/*F*_m_ ratio did not differ between the West and East orientation, thus suggesting that enhanced tocopherol contents in the West orientation served as a photoprotective strategy, as it has been previously shown in model plant species (Havaux et al., 2005). By contrast, melatonin contents did not increase in response to low temperature stress in the West-oriented individuals, despite exogenous applications of this compound have been shown to protect the photosynthetic apparatus from excess light stress, either caused by drought (Fleta-Soriano et al., 2017) or low temperatures (Ding et al., 2017). Inter-individual variability in melatonin contents was very high, as it occurred with leaf angles, which might mask a possible protective effect of these two mechanisms in populations growing under natural conditions. Indeed, the endogenous contents of melatonin found (in the order of ng/g dry matter, even lower than those found for indole-3-acetic acid) in *C. albidus* leaves suggest a modulatory role of this compound in plants, rather than a direct function as an antioxidant, which is in agreement with previous studies (Fleta-Soriano et al., 2017, see also Arnao and Hernández-Ruiz, 2019).

**Figure 7:**
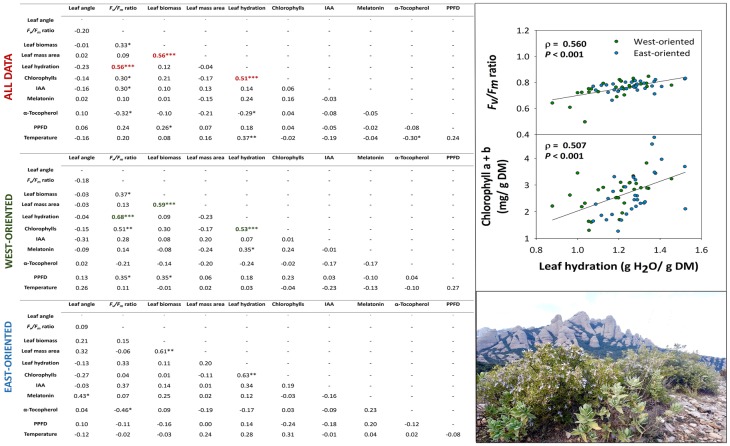
Relationship between all measured parameters in *C. albidus* from a natural population in the Natural Park of the Montserrat Mountains. Tables show the results of the Spearman’s rank correlation analyses (including the correlation coefficient, *ρ*, and *p* indicated with asterisks) for all data pooled together, for West-oriented individuals only and for East-oriented individuals only. All correlations were considered statistically significant at *p* ≤ 0.05, with one, two, and three asterisks indicating *p* below 0.05, 0.01, and 0.001, respectively. Correlations with a correlation coefficient, *ρ* ≥ 0.50, are shown in red, green, and blue for all data pooled together, West-oriented individuals, and East-oriented individuals, respectively. The plots in the right side of the figure show a graphical representation of the most biologically significant correlations. IAA, indole-3-acetic acid; PPFD, photosynthetic photon flux density.

Correlation analyses revealed interesting relationships between various parameters in the natural population, both when the analysis included all data and the East and West individuals separately (Figure 7). In the natural population from the Montserrat mountains, leaf angle did not correlate with any of the other studied parameters but the *F*_v_/*F*_m_ ratio correlated very significantly, strongly, and positively with leaf hydration (*ρ* = 0.68, *p* < 0.001), which might indicate a prevalent occurrence of increased photoinhibition in the more water-stressed leaves. Small reductions in leaf hydration, which may be caused by chilling stress in the West-oriented individuals, could explain this result. This correlation was confirmed when all data were pooled together, although the relationship was more moderate (*ρ* = 0.56, *p* < 0.001). Furthermore, a moderate, but very significant, positive correlation was found between leaf hydration and total chlorophylls (*ρ* = 0.51, *p* < 0.001) pooling all data together that were consistent in both West and East-oriented individuals (*ρ* = 0.53, *p* < 0.001; *ρ* = 0.63, *p* < 0.001, respectively). Simultaneously, the relationship between *F*_v_/*F*_m_ ratio and α-tocopherol was also significant, although not too strong, in both orientations but this time being negative (*ρ* = −0.32, *p* < 0.05; *ρ* = −0.46, *p* < 0.05, respectively). In this manner, given that leaf hydration was slightly lower and tocopherol contents were higher in the West-oriented individuals than in the East-oriented ones, α-tocopherol might be acting as an important photoprotection compound under abiotic stress conditions. Therefore, these results suggest that *C. albidus* plants activate two photoprotection mechanisms. First, reductions in leaf angle, which might serve as a first line of defense from winter photoinhibition in leaves, particularly occurring in the uppermost position of shoots; and second, increased accumulation of α-tocopherol, which might serve as a stress sensing and signaling mechanism to counteract combined high light and low temperature stress during winter. The increased accumulation of α-tocopherol appears to be particularly relevant when photoprotection by leaf orientation is minimal and leaves consequently require more sophisticated biochemical, photoprotection mechanisms. In this study, higher levels of α-tocopherol are observed under more stressful conditions (e.g., West-oriented individuals exposed to a combination of abiotic stresses) and when leaves occupy positions more distal to the top of the shoot, due to the developmental program, and they orient more perpendicular to sun’s rays.

It is concluded that *C. albidus* has very effective and fine-regulated photoprotection mechanisms, including (1) an adequate orientation of decussate leaves as part of the developmental program, in which the leaf angle is additionally modulated on a diurnal and seasonal basis, therefore contributing to prevent photoinhibition as a first line of defense and (2) enhanced tocopherol contents that may help withstand combined high light and low temperature stress in *C. albidus* growing under Mediterranean field conditions.

## Data Availability

All datasets generated for this study are included in the manuscript and/or the supplementary files.

## Author Contributions

MP-L and SM-B conceived and designed the experiment. MP-L, AC, and MM performed the experiments. MP-L analyzed the data. SM-B wrote the manuscript with the help of MP-L. All authors revised and approved the final manuscript.

### Conflict of Interest Statement

The authors declare that the research was conducted in the absence of any commercial or financial relationships that could be construed as a potential conflict of interest.
